# Photonics for Bioapplications: Sensors and Technology—2nd Edition

**DOI:** 10.3390/bios16100580

**Published:** 2026-10-16

**Authors:** Nélia Alberto, Maria Fátima Domingues, Nunzio Cennamo, Adriana Borriello

**Affiliations:** 1 Instituto de Telecomunicações, University of Aveiro, Campus Universitário de Santiago, 3810-193 Aveiro, Portugal; 2 Department of Bioengineering and Instituto de Telecomunicações, Instituto Superior Técnico, University of Lisbon, 1049-001 Lisbon, Portugal; fatima.domingues@tecnico.ulisboa.pt; 3 Department of Engineering, University of Campania “Luigi Vanvitelli”, Via Roma 29, 81031 Aversa, Italy; nunzio.cennamo@unicampania.it; 4 Department of Precision Medicine, University of Campania “Luigi Vanvitelli”, Via De Crecchio, 7, 80138 Naples, Italy; adriana.borriello@unicampania.it

The interaction of light with biological matter offers a unique combination of analytical capabilities, making photonics a powerful platform for sensing, imaging, and monitoring across a broad range of bioapplications. Optical approaches can provide access to molecular, biochemical, structural, and functional information while enabling measurements that are highly sensitive, minimally invasive, and, in many cases, compatible with real-time operation. This versatility has supported the use of photonic technologies in a wide range of applications, from molecular detection and biological monitoring to tissue characterization and point-of-care (PoC) analysis.

Progress in the field increasingly depends on bringing together advances at different levels of the sensing process. New photonic structures and functional materials need to be combined with appropriate recognition strategies, efficient light–matter interactions, reliable interrogation schemes, and robust methods for extracting meaningful information from optical signals. At the same time, moving from a promising sensing concept to a practical bioanalytical tool involves much more than achieving high sensitivity. Selectivity, reproducibility, stability, measurement conditions, and system integration all become important considerations, particularly when sensing is performed in complex biological environments or under conditions that are less controlled than those typically found in a laboratory setting.

This interaction among sensing principles, materials, device architectures, interrogation methods, and application requirements is shaping the evolution of photonic systems for bioapplications. Developments across different spectral regions and sensing modalities are providing complementary ways of improving analytical performance, while advances in miniaturization and integration are opening opportunities for more compact and application-oriented platforms. An important challenge is therefore not only to improve sensor performance but also to develop systems capable of providing reliable and meaningful information in realistic measurement scenarios.

The contributions collected in this Special Issue, “Photonics for Bioapplications: Sensors and Technology—2nd edition,” reflect many of these challenges and opportunities. The ten published studies span a diverse range of optical principles, materials, device concepts, and interrogation approaches, while addressing biological and biomedical applications at different stages of technological development. Together, they show how innovation in photonic structures and sensing mechanisms can be complemented by advances in sensing interfaces, instrumentation, and signal analysis to address specific bioanalytical needs.

Although the contributions explore different technologies and applications, several parallels can be drawn between them. Some studies focus on photonic structures and strategies for controlling or enhancing light–matter interactions, while others address sensing configurations, interrogation methods, and signal-processing approaches aimed at obtaining reliable information from optical measurements. These developments are complemented by studies directed towards molecular and biomarker detection, physiological monitoring, tissue characterization, and other biomedical applications. Other contributions explore real-time monitoring and PoC concepts, illustrating the broad range of settings in which photonic sensing technologies may be used.

In the following, we briefly introduce the individual contributions, highlighting their main developments and their role within the broader scope of this Special Issue.

For instance, García et al. review the use of plasmonic and dielectric metasurfaces to enhance spectroscopic techniques, with particular emphasis on surface-enhanced Raman scattering (SERS), surface-enhanced infrared absorption (SEIRA), and surface-enhanced fluorescence (SEF) (Contribution 1). By manipulating electromagnetic fields at the subwavelength scale, these engineered structures can significantly strengthen light–matter interactions. The review examines the underlying principles, different designs, advantages, limitations, and possible applications of both plasmonic and dielectric metasurfaces. Special attention is given to their ability to address some of the sensitivity and resolution limitations of conventional spectroscopy, highlighting their relevance for highly sensitive spectroscopic analysis and biosensing.

Moving into the terahertz region, Contribution 2 investigates an all-dielectric metasurface for biosensing based on multimode coupling. The proposed design combines magnetic dipole, toroidal dipole, and magnetic quadrupole modes to address the narrow bandwidth typically associated with conventional quasi-bound states in the continuum (QBIC) sensors. It provides broadband coverage of 0.42 THz, while maintaining a high-quality factor of 499, with angular variations below 16°. The observed behavior was further supported by an analytical model based on Maxwell equations and mode coupling theory. This combination of broadband operation and high-quality resonance could be valuable for the development of miniaturized THz biosensors aimed at molecular diagnostics and multi-analyte screening.

A different sensing strategy is presented by Abdossova et al., who developed a surface-modified semi-distributed interferometer (ESDI)-based sensor for refractive index measurements and biosensing (Contribution 3). The sensor incorporates a polydimethylsiloxane (PDMS) overlay modified with either gold films or gold nanoparticles, with the nanoparticle-coated configuration reaching an average refractive index sensitivity of 62.8 dB/RIU with a resolution of 3.9 × 10^−5^ RIU over the range of 1.34790–1.35981. When functionalized for vascular endothelial growth factor (VEGF) detection, it achieved a detection limit of 355 fM. These results show how surface modification can extend the same optical architecture from physical measurements to biological detection, making it an interesting option for compact and highly sensitive biosensing.

Optical fiber sensing is also explored in Contribution 4, where ball resonator and hybrid Fabry–Perot interferometer-ball resonator configurations were evaluated for monitoring glucose, temperature, and pH. Seven spectral demodulation methods were compared, among which the Karhunen–Loève transform (KLT) showed particularly robust performance. The hybrid configuration provided greater sensitivity to glucose, reaching 6.15 a.u./(mg/mL), whereas the ball resonator responded more strongly to temperature and pH variations. Beyond the sensor configurations themselves, this work underlines the importance of selecting an appropriate spectral processing method when dealing with complex sensor responses and multiparameter physiological monitoring.

Contribution 5 turns to nucleic acid detection through a DNA biosensor that combines upconversion nanoparticles (UCNPs) with porous silicon (PSi). Target DNA is immobilized within the porous substrate and hybridized with UCNP-labeled probe DNA. Upon excitation at 980 nm, the nanoparticles emit luminescence that can be related to the concentration of the target DNA, achieving a detection limit of 86 pM. The use of near-infrared excitation also helps reduce background fluorescence while providing favorable optical penetration. Together, these features offer a relatively simple and sensitive strategy for rapid nucleic acid detection.

In Contribution 6, silver nanocrystals are incorporated into an electrochemical immunosensor for prostate-specific antigen (PSA) detection. The nanocrystals were deposited onto a glassy carbon electrode, followed by immobilization of anti-PSA antibodies, while square-wave voltammetry was used for measurements over a PSA concentration range of 1–10 ng/mL. A detection limit of 1.14 ng/mL was obtained, together with good selectivity against potentially interfering substances, such as glucose, urea, and L-cysteine. Good stability and repeatability were also observed, supporting the use of this configuration as a sensitive platform for PSA detection.

The potential of Raman spectroscopy for dental applications is addressed in Contribution 7 through an optical fiber-based sensor designed for early caries detection. Raman spectral features associated with enamel mineral composition were used to distinguish between sound, affected, and carious enamel. Using parameters such as the carbonate-to-phosphate ratio and phosphate peak characteristics, a multinomial logistic regression model achieved an overall classification accuracy of 73%. The probe also maintained its performance under angular misalignments of up to 10°. By avoiding the complexity of conventional confocal Raman systems, this fiber-based configuration represents a promising route towards more accessible point-of-care dental assessment.

Contribution 8 examines a different challenge in optical biomedical sensing: the influence of specular reflection and overexposure on Mueller matrix polarimetry of biological tissues. These effects can lead to saturated image regions and distort polarization information during in vivo measurements. Experiments performed on porcine liver tissue using a 3 × 3 Mueller matrix allowed several polarization parameters to be evaluated under different reflection conditions. Some parameters showed greater resistance to errors associated with specular reflection and overexposure, providing useful information for improving the robustness of quantitative polarimetric imaging in clinical settings.

Real-time monitoring of cell cultures is the focus of Contribution 9, which explores white-light spectroscopy as a non-invasive method for following primary T-cell cultures, with potential relevance to CAR T-cell production. The method enables online optical monitoring without opening the culture system or causing sample loss. Primary T-cells displayed absorption spectra and growth dynamics that differed from those previously observed in established cell lines, with their expansion being better described by a power-law model than by simple exponential growth. Interestingly, the relationship between generation–time dynamics and the initial cell concentration suggests that these kinetic features could serve as an early indicator of the final culture concentration. This finding may support non-invasive monitoring strategies for advanced cell therapy manufacturing.

Finally, Contribution 10 explores plasmonic paper for colorimetric PoC sensing, paying particular attention to the conditions that influence its optical response. Transmission spectra of nanoparticle-containing plasmonic paper were correlated with RGB color signals obtained under different light sources. The analysis revealed that filtering properties and the light source significantly influence sensitivity and precision. This relationship between the plasmonic substrate and illumination conditions provides useful guidance for designing simple, low-cost, and user-friendly colorimetric PoC devices.

Overall, the contributions to this Special Issue illustrate the wide range of approaches currently shaping photonic sensing and its biomedical applications. From metasurfaces and nanostructured materials to optical fiber sensors, spectroscopy, polarimetric imaging, and electrochemical platforms, the collection brings together complementary strategies for improving sensitivity, reliability, and practical performance. Taken as a whole, these works provide a snapshot of recent progress in the field while pointing to new opportunities in biomedical diagnostics, monitoring, and point-of-care sensing.

As this Special Issue ends, we would like to thank everyone who contributed to its completion. We are especially grateful to the authors for sharing their research and bringing together different perspectives within “Photonics for Bioapplications: Sensors and Technology—2nd edition.” The range of topics represented in this collection reflects the evolving nature of the field and the growing role of photonic technologies in biosensing and biomedical applications. We also extend our sincere appreciation to the academic editors and reviewers for the time, expertise, and careful attention they devoted to the editorial process. Their constructive feedback and thoughtful evaluations were invaluable in helping authors strengthen their manuscripts and in ensuring the scientific quality of the Special Issue. We greatly appreciate their commitment and contribution to this collection.

